# Dietary EVOO Polyphenols and Gut Microbiota Interaction: Are There Any Sex/Gender Influences?

**DOI:** 10.3390/antiox11091744

**Published:** 2022-09-02

**Authors:** Massimo D’Archivio, Carmela Santangelo, Annalisa Silenzi, Beatrice Scazzocchio, Rosaria Varì, Roberta Masella

**Affiliations:** Gender-Specific Prevention and Health Unit, Centre for Gender-Specific Medicine, Istituto Superiore di Sanità, 00161 Rome, Italy

**Keywords:** extra virgin olive oil, polyphenols, gut microbiota, sex, gender, diet

## Abstract

Accumulating evidence indicates that regular consumption of extra virgin olive oil (EVOO), the main source of fat in the Mediterranean diet, is associated with beneficial health effects and a reduced risk of developing chronic degenerative disorders. The beneficial effects of EVOO can be attributed to its unique composition in monounsaturated fats and phenolic compounds that provide important antioxidant, anti-inflammatory, and immune-modulating activities. On the other hand, it is well known that the gut microbiota has several important roles in normal human physiology, and its composition can be influenced by a multitude of environmental and lifestyle factors, among which dietary components play a relevant role. In the last few years, the two-way interaction between polyphenols, including those in EVOO, and the gut microbiota, i.e., the modulation of the microbiota by polyphenols and that of polyphenol metabolism and bioavailability by the microbiota, has attracted growing attention, being potentially relevant to explain the final effects of polyphenols, as well as of the microbiota profile. Furthermore, sex and gender can affect dietary habits, polyphenol intake, and nutrient metabolism. Lastly, it has been recently suggested that differences in gut microbiota composition could be involved in the unequal incidence of metabolic diseases observed between women and men, due to sex-dependent effects on shaping gut microbiota profiles according to diet. This review summarizes the most recent studies on the relationship between EVOO polyphenols and the gut microbiota, taking into account possible influences of sex and gender in modulating such an interaction.

## 1. Introduction

The beneficial effects of polyphenols contained in extra virgin olive oil (EVOO) have been recognized by the European Food Safety Authority, which recommends a daily consumption of about 20 g of EVOO, containing at least 5 mg of hydroxytyrosol (HT) and its derivatives, to prevent the onset of cardiovascular disease (CVD) and inflammation and to counteract oxidative stress caused by free radicals [1]. Encouraging findings indicate that obesity, metabolic and digestive disorders, liver and neurological diseases, and cancer can also benefit from EVOO polyphenol ingestion [2], as demonstrated by the increasing number of clinical trials designed to understand and validate the benefits of EVOO and its polyphenols in human diseases, as seen in the public web archive of ClinicalTrials.gov: https://clinicaltrials.gov/ (accessed on 30 April 2022).

EVOO polyphenols exert their well-known health benefits by interacting with fundamental cell signaling and gene regulation pathways in target cells and tissues. These compounds, after ingestion, undergo biotransformation in the gut by the gut microbiota (GM) [3], now considered an essential ‘organ’ that contributes to the health status or, when altered (dysbiosis), to the onset and progression of diseases in humans [4,5]. The GM contains more than 1000 bacterial species and, to a lesser extent, viruses and archaea that participate in maintaining host homeostasis, in regulating digestion, metabolism, and immunity, and in the protection against pathogens [4]. Accumulating evidence indicates that GM composition can be modulated by dietary polyphenols, thus highlighting a two-way interaction between dietary polyphenols and GM in which host characteristics (sex, age, genetics and ethnicity) and external environmental factors (diet, lifestyle, antibiotics and drugs) can influence each other through complex mechanisms not yet fully elucidated [5,6].

However, although several data evidence that consumption of an EVOO-rich Mediterranean diet can have positive effects on GM and associated microbial metabolites [2,7], only a few human studies have evaluated the effect of EVOO polyphenols on the intestinal homeostasis. Additionally, the increasing awareness that sex (i.e., the sum of biological attributes such as chromosomes, gene expression, hormone levels, and function) and gender (i.e., the socially constructed roles, behaviors, lifestyle, expressions and identities strongly influenced by sociocultural factors) [8] influence body homeostasis and disease susceptibility [9], along with the interindividual variability in response to polyphenols intake [10], strongly suggests a sex/gender-specific approach in moving toward a science-based personalized nutrition.

The aim of this review is to present up-to-date information on how EVOO and its phenolic compounds influence the gut microbial composition according to sex and gender in order to gain a better understanding of the complex interaction between EVOO polyphenols and microbiota signatures. This knowledge has the potential to better define strategies aimed at preventing and/or delaying the onset of chronic degenerative disorders via appropriate gender/sex-specific dietary modifications.

We carried out a comprehensive literature search until 30 April 2022 using PubMed: https://pubmed.ncbi.nlm.nih.gov/ (accessed on 30 April 2022).

The search syntax was ‘extra virgin olive oil’, ‘EVOO polyphenols’, ‘tyrosol’, ‘oleuropein’, ‘hydroxytyrosol’ and ‘oleochantal’ in combination with ‘gut microbiota’ or ‘microbiome’. The scientific literature was searched for in vivo human and animal studies, reporting results on the interaction between EVOO polyphenols and the GM, including gender-based assessment.

## 2. EVOO Composition and Characteristics

Extra virgin olive oil is universally recognized as a symbol of the Mediterranean diet, and its effects on human health have been amply demonstrated by recent relevant intervention studies [11,12,13,14].

The credit for the beneficial properties of EVOO is due to its composition; EVOO contains high levels of monounsaturated acids (63–83%), mainly oleic acid, associated with a lower cardiovascular risk, and other valuable components such as phenols, phytosterols, tocopherols, and squalene, which albeit present in low percentages (1–2%) exert beneficial effects on health [15].

The lipid profile, a well-balanced ω6/ω3 ratio, and the presence of bioactive compounds have been linked to protective effects on coronary, autoimmune, and inflammatory disorders, as well as anti-thrombotic and regulatory effects of blood pressure [16].

From the fruit of the olive tree *Olea europaea* L., family Oleaceae, an oil is produced in which more than 30 hydrophilic phenolic compounds have been identified that contribute to the oil’s distinctive characteristic smell and flavor [17]. However, the content of these compounds depends on several factors, such as the olive cultivar, the stage of ripeness, certain environmental factors related to soil and cultivation practices, extraction conditions (heating, addition of water, and extraction systems used to separate the oil from the olive paste), and storage methods [18,19].

The cultivar, i.e., the variety of the olive, is the main differentiating factor in the quality of oils. Each cultivar has a specific organoleptic profile characterized by aromatic substances, number and contents of polyphenols, and specific composition of sterols [20]. Each cultivar also records differences in fatty acid composition. Furthermore, the production process of EVOO strongly influences the polyphenol content. Thus, this process plays a key role in the quality of the final product, giving final EVOO products with different concentrations of polyphenols ranging between 50 and 1000 mg/kg [16], most commonly between 100 and 300 mg/kg [21].

### 2.1. EVOO Polyphenols

Polyphenols are organic compounds that have aroused great interest for nutritional issues in recent decades. Several classes of polyphenols can be identified, namely, flavonoids, phenolic acids, phenolic alcohols, stilbenes and lignans, which differ in their chemical structure [22]. The classes of polyphenols are contained in different percentages in foods of plant origin, including EVOO [23].

In EVOO, the most abundant class is represented by secoiridoids that are present exclusively in plants belonging to the Oleaceae family and include oleuropein and ligstroside, as well as several isomers of oleuropein and ligstroside aglycons, such as the dialdehydic form of decarboxymethyl oleuropein aglycone (oleacein) and the dialdehydic form of decarboxymethyl ligstroside aglycone (oleocanthal). Lignans represent, after secoiridoids, another abundant class of phenolic compounds in EVOO; the most common representatives are pinoresinol and 1-acetoxypinoresinol. Another relevant class is that of phenolic acids, namely, hydroxybenzoic acid derivatives (such as gallic and protocatechuic acids) and hydroxycinnamic acid derivatives (such as caffeic and coumaric acids), and phenyl-alcohols, such as HT and tyrosol (Tyr). Flavonoids, luteolin and apigenin, are present in much lower levels than other phenols [24,25,26,27,28,29,30].

### 2.2. EVOO Polyphenols and Health

The scientific literature has provided evidence that polyphenols in EVOO contribute to the maintenance of human health by modulating metabolic processes, immune function, and cell proliferation [31,32]. The biological activity of polyphenols is strongly related to their antioxidant and anti-inflammatory properties, since they can improve the activity of the endogenous antioxidant system, reduce the pool of reactive oxygen species (ROS), neutralize potentially carcinogenic metabolites, and counteract the inflammatory processes associated with the onset of several pathological conditions [33,34,35]. For all these reasons, polyphenols, particularly EVOO polyphenols, have been considered as preventive and/or therapeutic agents against noncommunicable diseases (NCDs), such as CVD, type 2 diabetes, neurodegenerative disorders, and cancer, as well as obesity, a main risk factor for NCDs [31,32,36].

Several biological activities of polyphenols are closely linked to the antioxidant action they can exert at the level of cells and organs of the body. Oxidative stress, in fact, represents a common factor in the pathogenesis of several diseases [31,37,38]. However, emerging results suggest several mechanisms of action of EVOO polyphenols against oxidative stress that go beyond the conventional and direct free-radical-scavenging properties. In fact, EVOO polyphenols appear to be able to interact with cellular signals and influence gene expression, inducing an endogenous response to oxidative stress driven by modulation of different enzymatic activities [39,40].

Several studies have demonstrated that EVOO polyphenols are able to interact with the human immune system, influencing both the proliferation and the activity of lymphocytes and monocytes regulating the balance between anti- and pro-inflammatory cytokines, thus playing a role in inflammatory process control [41]. In particular, it has been suggested that the polyphenolic compounds in EVOO, once metabolized, can counteract the local and systemic inflammatory environment typical of immune-mediated inflammatory diseases) [33,42,43,44].

### 2.3. Biological Properties and Mechanisms of Action of Main EVOO Polyphenols in Preclinical Models

Among the EVOO polyphenols HT, Tyr, oleuropein, and oleocanthal have attracted great interest as a number of studies in preclinical models have demonstrated their protective power in various diseases including cardiovascular [27,45] and metabolic diseases [46].

#### 2.3.1. Hydroxytyrosol

The ability of HT to fight free radicals has been confirmed in some in vivo studies, underscoring a potential beneficial effect toward diabetes, both in terms of prevention and during therapy [47]. In vitro studies on the 3T3-L1 adipocyte cell line showed that HT is able to stimulate mitochondrial biosynthesis, which is usually reduced in diabetes mellitus [48]. The most reliable hypothesis is that HT acts through the upregulation of the peroxisome proliferator-activated receptor gamma coactivator-1 alpha (PGC-1α). In adipocytes, low concentrations of HT are able to increase the expression of all mitochondrial respiratory chain complexes, including ATP synthase. The presence of HT, therefore, protects mitochondria from reduced mitochondrial DNA synthesis by modulating the activity of critical transcription factors, such as nuclear respiratory factor 1 (Nrf1) and mitochondrial transcription factor A (Tfam) [48]. In addition, HT has been demonstrated to reduce epidermal growth factor receptor (EGFR) expression by promoting its degradation. This receptor is a key factor in colon carcinogenesis because it controls the balance between proliferation and apoptosis of cancer cells, as well as angiogenesis and metastasis growth [49]. HT has been shown to be an important cytotoxic agent in cellular models of breast cancer as it is able to inhibit the cell cycle in the G0/G1 phase through inhibiting cyclin D1 expression [50]. Furthermore, on the basis of the anti-inflammatory and antioxidant effects of HT, several authors have suggested a use of this compound in metabolic syndrome (MetS). In this regard, HT has been shown to influence the plasma metabolome, resulting in the reduction in unsaturated fatty acid biosynthesis and metabolism of linoleic and arachidonic acids, sphingolipids, and retinol, as well as in a better regulation of glycerolipid metabolism [51,52].

Lastly, again because of its antioxidant properties, HT is able to improve the health of the immune system. In this context, several studies have demonstrated the beneficial effects of HT in autoimmune diseases, particularly in rheumatoid arthritis, due to its ability to counteract inflammation and ameliorate oxidative damage by reducing the expression levels of proinflammatory cytokines and proteins through the JAK/STAT, MAPK and NF-κB pathways [53].

#### 2.3.2. Oleuropein

Like other phenolic compounds contained in EVOO, oleuropein exhibits antioxidant and anti-inflammatory properties and exerts protective actions at the cardiovascular, metabolic, neurological, and hepatic level [54,55,56,57,58]. Oleuropein appears to be a promising anti-inflammatory agent due to its ability to inhibit proinflammatory cytokine synthesis and lipoxygenase activity and/or to modulate inflammatory parameters [59]. In addition, it is able to modulate inflammation by downregulating key markers of inflammation pathways such as iNOS, COX-2, NF-κB and JNK, as well as the two proinflammatory interleukins, IL-6 and IL-1β [60].

This compound has been demonstrated the ability to reduce the number of blood vessels, showing antiangiogenic properties, as well as to inhibit LDL oxidation mediated by macrophages [61] and to protect the pancreatic β cells against the deleterious effect of cytokines [62]. In addition, the protective and therapeutic effects in several neoplastic conditions cannot be underestimated [63,64]. Indeed, oleuropein is able to modulate the tumor microenvironment under different conditions through its anti-inflammatory properties [65,66,67,68].

#### 2.3.3. Tyrosol

Tyr and its derivatives have shown antioxidant and protective activities toward circulating low-density lipoproteins (LDL) [69,70], albeit at concentrations much higher than for HT [65]. This effect is most likely due to the ability of Tyr to bind human LDL lipoproteins and to eliminate peroxyls in the arterial intima, where LDL oxidation mainly occurs. In this regard, by incubating plasma with EVOO extracts, a dose-dependent increase in the binding of phenolic compounds to LDL was observed. Tyr, as well as other phenolics, has also been evidenced after olive oil administration in postprandial studies, where plasma concentrations of Tyr and HT were directly correlated with changes in total phenolic compound content of LDL after ingestion of olive oil high in phenolic compounds [71]. An antiplatelet activity was also highlighted due to the modulation of cAMP phosphodiesterase and platelet-activating factor (PAF) [72].

The neuroprotective properties of Tyr were studied in vitro on cell lines and brain sections and in vivo in rat models of cerebral ischemia. In particular, Tyr showed a dose-dependent neuroprotective effect in a rat model of transient middle cerebral artery occlusion (2 h occlusion, 22 h reperfusion). Tyr reduced the infarct area in the treated group with respect to the untreated control group [73].

#### 2.3.4. Oleochantal

Oleocanthal, the compound responsible for the pungent and spicy taste of EVOO, has been studied for its anti-inflammatory and antineoplastic effects. The results of studies show, in fact, that this compound is able to inhibit cyclooxygenase (COX) 1 and 2 in a dose-dependent manner, imitating the anti-inflammatory action of synthetic nonsteroidal anti-inflammatory drug (NSAID) ibuprofen [74]. Furthermore, it is also able to inhibit COX 1 and COX 2 enzymes to a significantly greater extent than ibuprofen at equimolar concentrations. For example, oleocanthal (25 μM) inhibits 41–57% of COX activity in comparison with an equimolar concentration of ibuprofen, which inhibits 13–18% of COX activity [74]. Since COX 1 and COX 2 are responsible for the conversion of arachidonic acid into prostaglandins and thromboxanes, which are active inflammatory mediators, the attenuation of the activity these enzymes by oleocanthal produces an important anti-inflammatory response [75,76].

## 3. Gut and Sexual Dimorphism

It has become increasingly evident that GM is a fundamental factor in influencing human health and disease. This is because the huge number of interacting bacteria, archaea, and viruses that constitute the microbial communities present in the human gastrointestinal tract markedly influences host homeostasis in a mutual interaction [4]. The GM represents a dynamic structure continuously communicating with the environment. More than 1000 bacterial species can be found in the intestines; they can be categorized into several genera, families, orders, classes and phyla, and most of them belong to the phylotypes Firmicutes, Bacteroidetes, Actinobacteria, Proteobacteria, Fusobacteria, and Verrucomicrobia, mainly anaerobic species. Bacteroidetes and Firmicutes represent 90% of microbiota in the normal human gut [77]. This large and diverse microbial community contributes to human metabolism, participating in the metabolism of dietary carbohydrates, proteins, bile acids, phytochemicals, and plant polyphenols, as well as in the synthesis of essential amino acids and vitamins [4,6].

The gut microbiota, even though it shows great diversity of composition among individuals, exerts specific functions not only in the host metabolism of nutrients, but also in the maintenance of structural integrity of the gut mucosal barrier, immunomodulation, xenobiotic and drug metabolism, and protection against pathogens [5]. To ensure all these functions, a functional redundancy exists within the microbiota with different bacteria doing the same or similar activities [6].

The GM plays a major role in digesting food; on the other hand, the diet greatly affects the balance among microbial species. The gut microbiota helps to digest food and to extract nutrient and energy from it. In particular, the ingested dietary fibers are converted into short-chain fatty acids (SCFAs), such as acetate, propionate, and butyrate, which represent an extra source of energy [5] and act as regulators of systemic metabolism [78].

To date, although no standard composition exists to define a healthy human microbiota, three enterotypes (clusters of bacteria grouped by functions), independent of age, gender, cultural background, and geography, have been defined. Each of these three enterotypes is characterized by abundant levels of one of three genera; enterotype 1 is rich in *Bacteroides* (phylum Bacteroidetes), enterotype 2 is rich in *Prevotella* (phylum Bacteroidetes), and enterotype 3 is dominated by *Ruminococcus* (phylum Firmicutes) [79]. Each enterotype is associated with a different diet; specifically, enterotype 1 is associated with a diet rich in animal proteins and fats, but low in fibers (Western diet). Enterotype 2 is associated with a diet rich in carbohydrates, resistant starch, and fibers. Enterotype 3 is associated with long-term fruit and vegetable consumption [80]. An unbalanced diet may lead to altered gut bacterial composition (dysbiosis) and to the progression and development of metabolic and inflammatory diseases, such as diabetes, obesity, cancers, allergies, immunologic disorders, and infections [4].

The interindividual GM variability starts at birth and is influenced by birth delivery and breastfeeding, as well as throughout life by age, host genetics, ethnicity, and external environmental factors such as antibiotics, diet, lifestyle, and drugs. Only recently has sex been considered a significant factor contributing to GM variability [77,81,82]. In this regard, the close relationship between the host and microbiota also involves hormones. Indeed, the reciprocal interaction between the hormones produced by the host and those produced by the microbiota influences, in a sex-specific manner, hormonal signaling, governing not only appetite and metabolism [83,84,85] but also immune response [86], behavior, and stress response via the ‘gut–brain axis’ [87,88,89].

These findings suggest that the GM can be involved in the different sex-related predisposition to the diseases [90]. Indeed, the significant and reciprocal impact of sex hormones on GM led to the generation of the new term ‘microgenderome’. This refers to sex-specific microbiota and its bidirectional interaction with sex hormones and immune systems, and it represents an emerging research field in the study of sex-biased disease susceptibility [91].

The earliest study on the analysis of sex-related microbiota was conducted in 230 healthy subjects from four European countries (France, Germany, Italy, and Sweden) in 2006. The results showed that the microbial community in the fecal microbiota displayed sex-specific differences. Specifically, males appeared to have higher abundance of genera *Bacteroides* and *Prevotella* compared with females [92]. Data from 82 subjects recruited from Washington, D.C., area hospitals confirmed the lower abundance of Bacteroidetes in women’s GM compared to men’s, and this abundance was further reduced in overweight and obese women; no significant relationship between BMI and GM was observed in men. Moreover, the authors observed that the source of fibers was associated with different microbial taxa; fiber from beans was associated with an abundance of *Clostridia* (Firmicutes phylum) in women, and fiber from fruits and vegetables was associated with the Actinobacteria phylum in men [93]. Microbiota analysis of 75 Spanish subjects resulted in a lower abundance of *Bacteroides* in men, which further decreased as BMI increased. In addition, regardless of BMI, a higher content of *Veillonella* (Firmicutes phylum) and *Methanobrevibacter* (Euryarchaeota phylum), along with a lower abundance of *Bilophila* (Proteobacteria phylum), was observed in men compared to women. It was observed that the Firmicutes/Bacteriodetes ratio was higher in men with BMI <33 kg/m^2^ and lower when BMI was >33; this was not evidenced in women. It is known that body fat distribution also displays gender-specific differences related to sex hormones; thus, the authors suggested that the grade of obesity influenced microbiota differences in a sexual dimorphic manner [94].

Healthy Japanese subjects (*n* = 277; aged 20–89 years) showed significant differences in GM composition between males and females. Genera *Prevotella, Megamonas* (Firmicutes phylum), and *Fusobacterium* (Fusobacteria phylum) were higher in males, while *Megasphaera* and *Bifidobacterium* (Actiniobacteria phylum), as well as *Ruminococcus* and *Akkermansia* (Verrucomicrobia phylum), were higher in females. In addition, the authors observed that the consistency of stools was associated with different microbes. In males, Fusobacteria was significantly higher in the loose consistency group, while genus *Oscillospira* (Firmicutes phylum) was significantly higher in the hard consistency group; in females, genera *Campylobacter* (Proteobacteria phylum), *SMB53* and *Turicibacter* (Firmicutes phylum) were significantly higher in the hard consistency group [95]. Moreover, an interesting study conducted on 131 Spanish subjects showed that menopausal status and obesity reduced GM differences between men and women [96]. Specifically, the authors identified 273 different taxa between premenopausal women and men but only 103 between postmenopausal women and men. In addition, no differences were observed in the microbiota functionality between postmenopausal women and men, which was instead shown when the microbiota from premenopausal women and men were compared. In particular, premenopausal women had higher abundance of genera *Alistipes* (Bacteroidetes phylum), *Bifidobacterium* and *Ruminococcus*, but lower levels of *Bacteroides*, *Prevotella* and *Haemophilus* (Proteobacteria phylum) compared to men and postmenopausal women. The different hormonal status between pre- and postmenopause women suggested a masculinization of the GM composition after menopause. Moreover, the functional differences in GM observed between pre- and postmenopausal women disappeared in obese postmenopausal women, since adipose tissue replaced the estrogen synthesis by the ovary [96].

A study conducted in a cohort of 158 subjects with MetS (79 women and 79 men) from the CORDIOPREV study showed a differential microbial community composition according to sex. Fecal sample analysis showed a diverse abundance of microbes related to T2D and insulin resistance. Specifically, higher contents of genera *Collinsella* (Actinobacteria phylum), *Alistipes, Anaerotruncus* (Firmicutes phylum), and *Phascolarctobacterium* (Firmicutes phylum) were observed in MetS women than in MetS men, whereas *Faecalibacterium* (Firmicutes phylum) and *Prevotella* were higher in MetS men with respect to MetS women. This finding may support the influence of GM on incidence of metabolic diseases that results different between genders [90].

A recent study on fecal samples from 54 healthy Chinese students (aged 20–30) suggested that the diversity of bacterial community between males and females might be useful in forensic personal identification. Although no α diversity (diversity or richness of species within each sample) was revealed between sexes, it was observed that males had an abundance of Proteobacteria and females had an abundance of the Synergistetes phylum. In addition, in males, the Patescibacteria phylum increased in the underweight group while Bacteroidetes increased in the normal-weight group; in females, Bacteroidetes increased and Actinobacteria decreased in underweight subjects, while Firmicutes increased in overweight ones [97].

Taken together, all the above studies indicate a sex-related gut microbial community that should be considered in the future studies aimed to elucidate whether modifications in the composition of the GM are the cause or the consequence of different responses to different environments according to sex and gender.

## 4. Bioavailability of Polyphenols and the Two-Way Interaction with Gut Microbiota

Polyphenols contained in foods are generally conjugated with sugars or organic acids or are present as unconjugated oligomers. Following their ingestion, small amounts of polyphenols (about 5–10%) [98], mainly those with simple monomeric and dimeric structures, may be absorbed in the small intestine, via hydrolyzation of glycosides [22]. More complex polyphenols, especially with oligomeric and polymeric structures, cannot be absorbed in the small intestine; they, thus, reach the colon, where their structures are extensively modified. In particular, the intestinal microbiota first hydrolyzes glycosides into aglycones and then metabolizes them to simple phenolic acids [99,100]. Colon bacteria substantially contribute to the biotransformation of the polyphenols, breaking down unabsorbed compounds into a wide range of metabolites. Bacteria may also further modify enterocyte-derived metabolites [101].

This activity is of great importance for the biological action of these compounds, since it produces active metabolites. Prior to passage into the blood stream, the polyphenols, which are now simple aglycones, undergo conjugation processes [102]. Conjugation includes three different processes: methylation, sulfation, and glucuronidation. Then, polyphenols are distributed to organs and excreted into the urine [103].

Therefore, it is clear that the polyphenols are extensively modified after their ingestion, and any single polyphenol can generate several metabolites [104]; all these modifications deeply affect their biological activity [105,106]. It must be taken into account that the bioactive compounds present in cells and tissues are chemically, biologically, and functionally different from the ingested form.

Many studies suggest a two-way interaction between dietary polyphenols and the gut microbiota. It is well known that the GM has several important roles in normal human physiology. The composition of GM can be influenced by a multitude of environmental and lifestyle factors, among which diet [107,108,109], giving rise to dysbiosis, could play a key role in human disease progression.

Dietary polyphenols have been evidenced in numerous reports with health-promoting functions, but it has to be underlined that their beneficial effects are related to their bioavailability, which is dictated by the composition of the gut microbiota. Recent improvements in deep sequencing technologies and bioinformatics have enabled a more complex understanding of the reciprocal interactions of dietary polyphenols and gut microbiota, as well as their metabolic impact. This two-way interaction gives rise to two different phenomena. First, as previously described, the GM is able to metabolize polyphenols, yielding many active metabolites [110,111,112]. Polyphenols and their metabolites may act on metabolic pathways and confer health benefits [113,114]. Accordingly, the possible beneficial effects of polyphenols are related not only to their dietary intake, but also to the individual capacity of metabolizing them [112], clearly highlighting that a different composition of the GM will cause a different biotransformation of dietary polyphenols.

On the other hand, polyphenols may directly modulate the gut microbiota. Recent studies suggest a prebiotic-like effect of polyphenols. Prebiotics are defined as substrates selectively utilized by the host’s microorganisms, resulting in benefits for metabolic health, gastrointestinal system, bone health, and mental health [115]. Polyphenols are indeed able to stimulate the growth of beneficial bacteria, such as the genera *Lactobacillus* spp. (Firmicutes phylum), *Bifidobacterium* spp., *Akkermansia* spp., *Roseburia* spp. and *Faecalibacterium* spp., which provide antipathogenic and anti-inflammatory effects and cardiovascular protection [116,117]. Moreover, they are also able to hamper the increase in pathogenic bacteria, such as *Clostridium* spp. (Firmicutes phylum) [118,119]. Human in vivo studies have confirmed that polyphenols are able to remarkably shift the ratio between beneficial and harmful bacteria in gut microbiota, increasing the beneficial bacterial strains [120,121].

In this context, EVOO and its phenolic compounds, acting as prebiotics, can stimulate the growth of beneficial bacteria, increase the production of microbial-produced SCFAs, and reduce the abundance of pathogenic bacteria [7]. Specifically, EVOO ingestion enhances the growth of *Lactobacillus* and *Bifidobacterium*, which are also SCFA-producing bacteria, associated with potential anti-obesity effects in humans [122,123].

Hence, this section discusses the two-way synergistic interactions of EVOO dietary polyphenols on the gut microbial composition.

### 4.1. Gut Microbiota Metabolizes EVOO Dietary Polyphenols

As previously described, the most representative microbiota phyla are Firmicutes, Bacteroidetes, Proteobacteria, Actinobacteria and Verrucomicrobia [124]. Bacteroidetes (such as *Prevotella* and *Bacteroides* genera) and Firmicutes (such as *Clostridium, Enterococcus, Lactobacillus,* and *Ruminococcus* genera) generally represent more than 90% of bacterial species in healthy people [125]. Evidence has been provided about the influence of diet on the composition of GM, which can impact health, with either beneficial or deleterious effects. For instance, *Prevotella* is the main bacteria in the gut microbial community of people eating carbohydrate-rich diets, whereas *Bacteroides* is the predominant one in people following diets rich in animal protein and saturated fat [126,127]. Within a Mediterranean type of diet, a good portion of ingested polyphenols derives from EVOO, and most of them undergo an extensive gastrointestinal biotransformation, yielding active metabolites. Since the various microbiota phyla differently metabolize the dietary components, differences in GM composition may lead to different bioavailability of nutrient metabolites. The individual’s ability to produce specific metabolites is consequently associated with different ‘metabotypes’ [128,129].

EVOO polyphenols are significantly absorbed (40–95%) in a dose-dependent manner in humans [71,130,131,132], and the major site for the absorption of these compounds is the small intestine [130,133]. They reach the colon in the parenteral form or are partially modified in the acidic environment of the stomach, releasing free phenolic alcohols [134,135]. Most of the studies regarding the metabolic fate of EVOO polyphenols after ingestion have focused on three of the most abundant ones: HT, Tyr and oleuropein [136,137].

Oleuropein aglycone and its dialdehydic form are likely not absorbed as such in the small intestine [135]. On the other hand, if the ingested oleuropein is glucosylated, it is not to subjected to gastric hydrolysis [130], entering the small intestine unmodified, along with high amounts of free HT and Tyr.

Lactic acid bacteria, as *Lactobacillus* and *Bifidobacterium* species, which contribute, as probiotic bacteria, to maintain or improve microbial balance in the gut [138], efficiently degrade oleuropein. In particular, *Lactobacillus plantarum* is effective in converting oleuropein into HT [139,140,141,142] because of β-glucosidase and esterase activities. In addition, it metabolizes several phenolic acids such as protocatechuic ferulic, gallic, and coumaric acids [142].

Corona et al. conducted one of the first studies on the biotransformation of ingested EVOO polyphenols by the colonic microflora [134]. The authors, using human fecal microbiota and a perfused rat intestinal model, were able to demonstrate that both HT and Tyr are extensively metabolized in the gastrointestinal tract to be eventually absorbed as simple phenols in the small intestine. On the other hand, oleuropein reached the large intestine as an unmodified compound, and then it was rapidly degraded by the colonic microflora to yield mainly HT.

Using the same in vitro experimental model, Mosele et al. [143] demonstrated that oleuropein was rapidly de-glycosylated during 6 h of incubation by the human fecal microbiota, and then degraded into elenolic acid and HT by microbial esterases, disappearing after 48 h. On the contrary, HT constantly increased during the fermentation period, representing the main product of oleuropein microbial metabolization. All these results were confirmed in vivo by the same authors, showing that a 3 week intake of EVOO polyphenols significantly increased the concentration of HT in the feces of human volunteers.

The most comprehensive study regarding the identification of metabolites in human urine of the main EVOO polyphenols (i.e., secoiridoids, flavonoids and phenolic alcohols) was conducted by García-Villalba et al. [144]. The authors identified about 60 metabolites, with the largest number produced from HT, oleuropein aglycone, and oleocanthal. Conversely, the lowest number of metabolites came from Tyr, luteolin, apigenin, pinoresinol and acetoxypinoresinol, suggesting that these compounds may have been excreted in feces, destroyed in the gastrointestinal tract, or poorly absorbed [144].

However, data on the microbial change of oleuropein and other phenolics from EVOO in humans are still scarce, and conflicting results have been reported on the level and the forms found in different biofluids [122,145].

In an in vivo study with olive leaf extract administration in nine volunteers, a large interindividual variation in absorption and metabolism of EVOO polyphenols was highlighted, possibly resulting from differences in human enzymatic activity. Interestingly, the authors noted a sex effect, with males more efficient at conjugating oleuropein [146].

All these metabolic modifications do not inhibit the biological activities of polyphenols [147,148]; the yielded metabolites and the parent compounds are both capable of reaching a sufficient concentration at the tissue level to exert their antioxidant and anti-inflammatory actions, by modulating intracellular signaling [24].

Overall, these results demonstrated that GM had a profound impact on the biotransformation of EVOO dietary polyphenols, also showing the huge potential of the metabolites produced by the intestinal microflora as promising substances for the prevention or treatment of many diseases. These findings clearly highlighted how the different composition of the microbiota among individuals can cause the different biotransformation of EVOO dietary polyphenols [149]. Accordingly, the beneficial effects depend not only on the dietary polyphenol taken from the diet, but also on the type of microbial population of the individual.

Therefore, future studies on human volunteers are needed to provide a basis for gut microbiota-based therapeutic applications of dietary polyphenols.

### 4.2. EVOO Dietary Polyphenols Modify Gut Microbiota

Dietary polyphenols and their metabolites may strongly influence microbiota composition, inhibiting the growth of harmful bacteria and exerting prebiotic-like effects toward beneficial bacteria [101].

The mechanisms via which these compounds can modulate the GM need to be fully highlighted, but probably involve either direct or indirect mechanisms. Indeed, polyphenols can directly stimulate or inhibit bacterial growth, with the inhibition referring to the bactericidal or bacteriostatic effect of phenolic compounds toward potentially pathogenic bacteria. It is important to note that the concentration and characteristics of polyphenols must be considered. On the other hand, polyphenols can indirectly affect the growth of one group of bacteria by increasing the development of another group [150,151].

Polyphenols exert a prebiotic action on specific strains, enhancing the abundance of beneficial bacteria such as the genera *Bifidobacterium* and *Lactobacillus*, which contribute to gut barrier protection, *Faecalibacterium prausnitzii,* which presents anti-inflammatory action by blocking NF-κB activation, and *Roseburia* spp., which are butyrate producers [152]. Romero et al. clearly demonstrated that EVOO polyphenols could spread in gastric juice, surviving for several hours in the acidic environment, and they could exert a bactericidal effect against eight different strains of *Helicobacter pylori* at very low concentrations (1.3 μg/mL) [153]. These results highlighted the possibility of using EVOO as a chemopreventive agent for ulcer and/or gastric cancer.

The modulation of GM by oleuropein was recently confirmed by in vitro metagenomic sequencing [154]. Phenolic leaf extracts obtained from olive leaves of two different cultivars were subjected to in vitro fecal fermentation. A commercial EVOO sample was used as a reference. As expected, the in vitro processes decreased oleuropein content and determined a concurrent increase of HT and other phenolic metabolites. Interestingly, metagenomics sequencing of microbiota revealed differences in modulation, at both the family and the genus level, between the leaf extract and EVOO, highlighting that high Coriobacteriaceae content was found in leaf extract samples, while Lactobacillaceae was more abundant in EVOO samples.

Thus, oleuropein possesses prebiotic properties, and this is also due to the fact that *Lactobacillus* and *Bifidobacterium* strains may utilize oleuropein as a carbon source [134]. Oleuropein is also effective in *E. coli* growth inhibition [155], similarly to HT, which exhibited a significant antimicrobial activity against selected *Enterobacter* species [156]. Several experimental trials showed oleuropein and HT to represent the best inhibitors of several gastrointestinal pathogens, as reported by Thielmann et al. [157].

These findings support the modulation of the GM exerted by EVOO polyphenols. Unfortunately, this great amount of data arises from in vitro experiments and may not mimic the in vivo conditions.

Pallara et al. evaluated the profile of PUFAs derived from ruminant livestock [158] after the administration of feed supplemented with stoned olive pomace, a waste deriving from the processes of conversion from olive to olive oil. Their results demonstrated that feeds supplemented with stoned olive pomace decreased the production of unsaturated FA in a dose-dependent manner through the modification of GM composition.

Another animal study was performed to compare the effects of standard diets vs. high-fat diets enriched with butter, EVOO, or refined olive oil on GM composition [159]. The group fed with a high-fat diet enriched with refined olive oil showed not only significant higher levels of total cholesterol compared to the EVOO group, but also a greater presence of Desulfovibrionaceae (Proteobacteria phylum), Spiroplasmataceae (Tenericutes phylum), and Helicobacteraceae (Proteobacteria phylum) families. The authors showed a direct relation between the quality of dietary fats and the presence of certain taxa, clearly highlighting that that the minor polar compounds present in EVOO were able to positively modulate the GM composition. Moreover, Prieto et al. [160] confirmed that the intake of EVOO polyphenols influenced the composition of the mouse intestinal microbiota, positively affecting the health status.

In spontaneous hypertension rats, Hidalgo et al. [161] investigated the effects of EVOO on GM composition. After 12 weeks, rats fed for 12 weeks with a diet enriched with EVOO showed significant differences in *Lactobacillus* and *Clostridia XIV* percentage with respect to rats fed with a standard diet.

To the best of our knowledge, human studies specifically regarding the impact of dietary intake of EVOO polyphenols on the microbiota are still scarce.

In a randomized double-blind human trial involving 12 hypercholesterolemic adults, changes in fecal microbial populations were evaluated following sustained consumption of EVOO polyphenols, alone or in combination with thyme polyphenols [162]. A significant increase in the *Bifidobacterium* group was detected when a mixture containing EVOO and thyme polyphenols was ingested. The stimulation of *Bifidobacteria*, together with the increases in microbial phenolic metabolites with antioxidant activities, such as protocatechuic acid and HT, exerted a cardioprotective effect in hypercholesterolemic subjects [162]. The same study also reported a slight HT modification in microbial composition following EVOO polyphenol intake, depending on the dosage, as confirmed in a randomized crossover human study on 10 hypercholesterolemic subjects [3]. The effects of the consumption of 25 mL/day of different olive oils for 3 weeks on human intestinal immune function were evaluated. The consumption of olive oil with a high content of phenolic compounds induced a change in the intestinal mucosa composition, thus increasing the stimulation of the intestinal immune system.

In a dietary intervention, 62 healthy hypercholesterolemic patients were randomly assigned to eat 90 g of olive pomace-enriched biscuit or an isoenergetic diet for 8 weeks. The authors reported changes within the composition of the gut microbiota, showing a small increase in *Bifidobacteria*, as well as an upregulation of microbial polyphenol biotransformation in the intestine [163]. A sex effect, possibly linked to differences in microbiota, was also found, with statistically significant differences in relative abundances of fecal bacteria between men and women.

An in vivo study on 18 overweight/obese subjects vs. 18 normal weight subjects showed that a Mediterranean diet enriched with 40 g/day of EVOO was able to modulate the composition of the gut microbiota, inducing an increase in lactic acid bacteria [123]. These bacteria include a large number of genera, such as *Lactobacillus*, considered beneficial for the host and implicated in the pathogenesis of obesity and cardiovascular diseases [164]. These results evidenced the role of GM as a key element in the regulation of energy homeostasis, supporting the potential role of EVOO polyphenol intake as a useful dietary intervention for decreasing inflammation and oxidative stress in obese patients.

The studies carried out so far clearly suggest that phenolic compounds and their metabolites can impact bacterial growth and GM diversity, consequently affecting GM activities at the local and systemic level. However, the complex interrelation between EVOO polyphenols and human microbiota is still far from being exhaustively investigated, and further studies are mandatory to provide consistent evidence.

## 5. EVOO Beneficial Effects on Gut Microbiota: Possible Differences between Sexes?

It has long been known that clinical response to drug administration varies among individuals and is influenced by sex, while lifestyle and dietary habits are strongly influenced by gender [165]. Consequently, high human interindividual variability has been observed in response to polyphenol consumption, with the contribution of several individuals’ conditions, including sex and gender, since they markedly influence phenol bioavailability, metabolism, and response to the intake. On the other hand, as discussed above, there is increasing evidence of the importance of the two-way interaction between polyphenols and GM on human health [166,167]. However, only very few data are available regarding sex/gender influences on the interactions between GM and EVOO polyphenols in humans. Unfortunately, the studies conducted in animals have also generally considered one sex at a time (i.e., male or female), hindering a real and effective evaluation of differences between males and females in the response to EVOO ingestion (Table 1).

EVOO phenolic compounds, such as HT, promote the growth of *Bifidobacteria*, which are in part responsible for the anti-inflammatory properties in the gut. Specifically, they produce an increase in Bacteroidetes or a reduction in Firmicutes/Bacteroidetes ratio, which is associated with atheroprotection [162]. The Firmicutes/Bacteroidetes ratio is a biomarker of altered colonic microbial composition [122] as also observed in HIV-infected patients that showed GM alterations closely associated with immune dysfunction. A 50 g EVOO daily ingestion for 12 weeks by HIV-infected Spanish patients over 50 years of age improved α diversity in the gut microbiota. In all subjects, it was observed that EVOO ingestion induced a decrease in proinflammatory families such as Dethiosulfovibrionaceae that is frequently increased in the microbiota of subjects affected by other chronic inflammatory conditions. Significant differences were observed between male and female HIV subjects in the effects of EVOO on microbiota; an increase in taxa *Prevotella*, Bacteroidetes, *Bifidobacterium*, Erysipelotrichaceae, and *Eubacterium* was observed in males, while *Eggerthella, Ruminococcus,* Lachnospiraceae, Parabacteroides, and *Akkermansia* were increased in women [169]. These changes in microbiota composition may have some effects on immune and inflammatory functions. *Prevotella*, indeed, participates actively in the modulation of the gut immune system and has been associated with an increase in activated CD4 lymphocytes and with higher plasma levels of proatherogenic metabolites [164], *Lachnospira* and *Ruminococcus* are associated with SCFA production, and *Akkermansia* is associated with the inflammatory response [168].

In a double-blind dietary intervention, 62 healthy hypercholesterolemic male and female subjects randomly consumed 90 g of olive pomace-enriched biscuit (olive-enriched product, OEP; containing 7.1 ± 4.01 mg/100 g of HT and its derivatives) or an isoenergetic diet for 8 weeks. Although ingestion of OEP biscuits did not change blood lipid profiles, it resulted in increased excretion, in men compared with women, of small phenolic acids (e.g., 3,5-diOH-benzoic acid, *t*-coumaric acid, naringenin, 4-hydroxybenzoic acid, and 4-hydroxyphenyl acetic acid), which are metabolites of olive polyphenols. Following dietary intervention, fecal sample analysis revealed greater abundance of taxa *Akkermansia, Bifidobacterium, Bacteroides* (Bacteroidetes phylum), Enterobacteriaceae (Proteobacteria phylum), Rikenellaceae (Bacteroidetes phylum), and Barnesiellaceae (Bacteroidetes phylum) in women, and of *Prevotella* and *Eubacterium* (Firmicutes phylum) in men. *Akkermansia* and *Bifidobacterium* are associated with protection from metabolic and cardiovascular disease, while *Bacteroides* and *Prevotella* are associated with protection from obesity. These data indicate that sex determines specific combinations of different dietary metabolites/gut microbial profiles useful to study sex-specific response to diet and, therefore, to design an appropriate dietary intervention [163].

Although most of the studies in animal models were not carried out in contemporary male and female animals, they clearly demonstrated that the administration of diet containing EVOO or olive derivatives induced different GM profiles favoring the proliferation of different bacteria taxa in male or female.

Prieto and coworkers fed male Swiss Webster ICR (CD-1) mice with three diets: a standard diet or two high-fat diets (35% total energy) enriched with butter (BT) or EVOO rich in polyphenols (527 mg/kg) for 12 weeks. Results showed a clear correlation between several variables involved in metabolic syndrome (blood pressure, insulin, diuresis, body weight and ghrelin) and specific gut bacterial taxa. Specifically, higher abundance of genus *Desulfovibrio*, BT-induced, positively correlated with high levels of systolic blood pressure. EVOO-fed mice exhibited lower levels of insulin and reduced abundance of *Desulfovibrio*; higher abundance of Proteobacteria Sutterellaceae and *Marispirillum* and Bacteroidetes *Mucilaginibacter dageonensis* correlated with low levels of plasmatic leptin. The authors hypothesized that the low levels of *Desulfovibrio* found in EVOO-fed mice was due to the high amount of polyphenols in EVOO [160].

Subsequently, the same authors, by adding a fourth experimental group fed with refined olive oil (ROO), reinforced the role of EVOO polyphenols in reducing *Desulfovibrio* in HFD-mice. Indeed, they observed a significantly higher abundance of *Desulfovibrio* (correlating with total cholesterol) in ROO-fed mice compared with those fed with EVOO. Moreover, the ROO-induced higher levels of genera from Spiroplasmataceae and Helicobacteraceae that correlate with total cholesterol, as well as the lower abundance of the above taxa detected in EVOO-fed mice, once again indicated the protective role of polyphenols [159].

Supplementation of obese male mice (HFD) with olive leaf extract (OLE, containing 12% (*w*/*w*) of phenolic compounds, in which oleuropein accounts for the 10%) for 5 weeks resulted in the improvement of glucose intolerance and obesity-associated inflammatory markers (Tnfα, Il-1β and Il-6, and Mcp-1), in liver and fat tissue. Obesity associated intestinal dysbiosis was ameliorated by OLE ingestion. OLE was able to reduce the increased Firmicutes/Bacteriodetes ratio, and to increase the abundance of the classes Actinobacteria and Bacteroidia, as well as of *Cytophaga* and *Akkermansia*, in HFD mice. Thus, OLE treatment by inducing gut modifications contributes to the modulation of the altered immune response in obese mice [169]. Another study investigated the effect of Chinese olive fruit (*Canarium album* L.) extracts (OE; containing 10 wt.% HT) on female mice treated with 100 mg/kg b/w OE for 4 weeks. The results indicated that OE increased the activity of the antioxidant enzymes superoxide dismutase (SOD), glutathione peroxidase (GSH-Px), and catalase (CAT) and decreased malondialdehyde (MDA) level in the serum, suggesting that OE treatment enhanced the antioxidant capacity in mice. OE ingestion reduced mRNA expression of SOD1, CAT, Gpx1 and Gpx2, as well as of proinflammatory cytokines TNFα and IL-1β, and it increased MDA in the intestine. OE supplementation enhanced the Firmicutes/Bacteroidetes ratio that positively correlated with serum GSH-Px activity and negatively correlated with MDA. In addition, a negative correlation was observed between *Alloprevotella* (Bacteroidetes phylum) and the serum total antioxidant activity (T-AOC), while *Colidextribacter* (Firmicutes phylum) was positively correlated with serum MDA and negatively correlated with serum T-AOC, SOD, and GSH-Px levels. Overall, these findings strongly connect the antioxidant capacity and anti-inflammatory activity of OE ingestion with the modification of Firmicutes and Bacteroidetes, differently involved in the modulation of inflammatory factors [170].

Supplementation with HT (50 mg/kg/day for 8 weeks) improved obesity, insulin resistance, and gut dysbiosis in male obese mice (HFD). HT positively modulated the expression of TLR4, TNFα, IL-1β, IL-6, p-JNK, IκBα, p-IRS and p-AKT in the liver, and modified the altered GM induced by high-fat diet (HFD) consumption decreasing the abundance of Ruminococcaceae, Proteobacteria, Ferribacter, Christensenellaceae (phylum Firmicutes), and Rikenella (phylum Bacteroidetes) [171]. Interestingly, Wang and coworkers examined the capability of HT to counteract the effects due to the exposure to air pollutant fine particular matter (PM2.5; ≤2.5 μM) in female adult C57BL/6J mice. For this purpose, mice were exposed to PM2.5 and supplemented with HT (50 mg/kg/day) for 4 weeks. The data obtained showed that HT reduced the visceral adipose tissue (VAT) and stimulated the energy metabolism of brown adipose tissue (BAT) and subcutaneous white adipose tissue (e.g., by reducing PPARγ and CEBPα, and by increasing UCP1 and phospho-AMPK) in PM2.5-exposed mice. Moreover, oxidative stress, NF-κB-associated hepatic inflammation, and insulin signaling were improved by HT treatment. These responses were associated with changes in the gut bacterial community; HT reduced Firmicutes and Actinobacteria (both associated with obesity) abundant in PM2.5-treated mice, and increased Bacteroidetes. A more accurate analysis of the gut microbiota revealed that HT increased Ruminococcaceae, Mycoplasmataceae (beneficial for glucose metabolism), and Erysilpelotrichaceae (Firmicutes phylum), as well as Prevotellaceae UCG-001 and Porphyromonadaceae (Bacteroidetes), decreased by PM2.5 exposure. HT also maintained gut homeostasis by increasing the abundance of *Akkermansia*, a well-known beneficial Verrucomicrobia and by reducing *Bifidobacterium*. All these findings showed the ability of HT to modulate the microbial ecosystem [172].

In addition, Tyr ingestion (0.2% *w*/*w* for 16 weeks) ameliorated the obesity condition in HFD-fed male mice. Tyr supplementation, indeed, reduced plasma triacylglycerol (TG), total cholesterol (TC), LDL-C, AST, ALT and fasting glucose levels, as well as body weight, fat weight, and adipocyte size, in HFD mice. Tyr changed the GM in mice; it modified the HFD-induced imbalance of the Firmicutes/Bacteroidetes ratio (e.g., by reducing Firmicutes abundance) and increased the abundance of Verrucomicrobia. Specifically, Tyr increased the genera *Colidextribacter* and *Oscillibacter* and the specie Lachnospiraceae *Bacterium 28_4,* and decreased the genus *Lactobacillus* and the specie Lachnospiraceae *Bacterium DW59*. In addition, Tyr increased the expression of genes related to thermogenesis (e.g., UCP1, UCP2, PGC-1α, PRDM16, CPT1β, DIO2 and EOLVL3), suggesting its involvement in reducing adiposity in both brown and inguinal white tissues. Interestingly, modification in GM correlated with metabolic parameters since *Colidextribacter* and *Oscillibacter* positively correlated with thermogenic genes and negatively correlated with TC, TG, and weight of WAT, thus indicating that Tyr might act as a potential prebiotic, by modifying the GM to influence adipocyte function [173].

An interesting study, using a single source of fat in the diet to induce obesity, e.g., 60% kcal from fat of coconut, sunflower, or EVOO (372 mg/kg polyphenols), for 16 weeks in female CD1 mice showed that even if the three HFDs induced gut dysbiosis, EVOO-HFD caused GM modifications associated with colorectal cancer (CRC) prevention. Specifically, EVOO-HFD increased the Firmicutes/Bacteroidetes ratio (this ratio correlates with obesity, as well as CRC prevention), significantly decreased *Bifidobacterium*, and maintained the abundance of *Akkermansia muciniphila* (associated with health benefits), unlike other diets. EVOO-HFD significantly decreased the abundance of opportunistic pathogens such *Enterococcus*, *Staphylococcus*, *Neisseria*, and *Pseudomonas* spp., thus creating an anti-inflammatory microenvironment [174].

In another study carried out in male mice, Millman and coworkers investigated the effects of EVOO ingestion on gut microbiota, mucosal immunity, and metabolic health in HFD-mice. For this purpose, male C57BL/6J mice were fed with low fat (LF; 10% energy from fat), high saturated fat (HFD; 45% energy from fat, 35% from lard), high fat/extra virgin olive oil (HFD-EVOO; 45% energy from fat, 35% from EVOO), and high fat/flaxseed oil (HF-FO; 45% energy from fat, 35% from FO) for 10 weeks. The results showed that HFD-EVOO and HFD-FO were able to increase microbial diversity, to reduce the abundance of Firmicutes, and to induce lower blood glucose levels, in HFD mice. Moreover, higher abundance of *Mucispirillum* (Deferribacteres phylum), Lachnospiraceae (Firmicutes phylum), and *Bacteroides* was observed in HF-EVOO compared to LF mice, as well as of *Allobaculum* (Firmicutes phylum) compared to HF. The authors hypothesized that phenolic compounds present in EVOO may act as prebiotics stimulating the growth of beneficial bacterial species and influencing microbial diversity. In addition, EVOO increased the mRNA expression of the transcription factor FoxP3 and IL-10, an inducer of regulatory T cells (Treg), in the intestines of HF mice, indicating its involvement in the maintenance of epithelial homeostasis in the gut. Indeed, the elsewhere reported observation that *Mucispirillum,* Lachnospiraceae, and *Bacteroides* are capable of increasing Treg indicated the complex and pleiotropic activity of EVOO in preserving immune metabolic health in mammals [175].

Lastly, another study evaluated whether oleuropein could ameliorate the advanced stage of T2D by modulating the composition and function of GM. Male diabetic db/db mice treated with oleuropein (200 mg/kg for 15 weeks) showed reduced fasting blood glucose and HOMA-index, as well as activation of hepatic insulin signaling by reducing the protein tyrosine phosphatase 1B (PTP1B) and increasing phospho-Akt. Feces sample analysis showed that oleuropein increased the abundance of Verrucomicrobia (*Akkermansia*) and Deferribacteres phyla, as well as reduced Bacteroidetes (*Prevotella*, *Odoribacter*, and *Parabacteroides*) and *Ruminococcus* [176]. Altogether, these data confirmed results elsewhere obtained showing that oleuropein-induced modifications of the above microbe community are associated with T2D improvement.

## 6. Conclusions

This review summarized the current knowledge about the relationship between GM and EVOO phenolic compounds, while also trying to highlight a possible role of sex/gender. Unfortunately, very few data are available in humans, and animal studies very often considered only one sex at a time, hindering a correct evaluation of differences between males and females in response to the ingestion of EVOO polyphenols. Moreover, it is necessary to take into account that translating data from laboratory animals to humans is very difficult, due to possible differences in nutrient metabolism and microbial composition, as well as to diverse responses to environmental factors, especially to the diet.

Nevertheless, the data we collected clearly confirm that the consumption of EVOO polyphenols has a beneficial effect on GM, promoting the growth of beneficial bacteria in both sexes. In addition, some studies, although limited in number, suggested that some differences in the taxa that are modified may occur between the two sexes.

However, the human GM composition is highly complex, and the relative proportion of bacterial types varies widely among individuals due to the vast number of interfering variables that affect the individual response to polyphenol consumption. In addition, one has to be aware that significant differences in metabolite concentrations may be observed, even in subjects consuming the same diet. All these things considered, it should be taken into account a possible different sex/gender response to EVOO polyphenol intake, and more human randomized trials, enrolling both men and women, are mandatory to establish a causal role among dietary polyphenols, specific GM ecologies, and health effects.

Gut microbiota may be influenced by age, ethnicity, dietary habits, and use of xenobiotics (e.g., oral contraceptives). All these factors, related to both sex and gender, might interact each other and provide different effects depending on their combination. This review was aimed at highlighting the complexity of these relationships. Women represent, of course, ‘a complex system’, as they show relevant changes in their physiology throughout their life course. In addition, other factors such as dietary habits might be strongly influenced by sociocultural factors, differently affecting women and men.

Unfortunately, the role of sex/gender in the management of health and diseases has been underestimated, and we are strongly convinced about the importance of integrating sex/gender analysis in future research, as required now by the most important funding agencies, such as the European Commission and the US National Institutes of Health.

A better knowledge of the interaction between GM composition and EVOO polyphenols will provide opportunities for the development of nutritional advice to modulate GM and, by taking advantage of the prebiotic-like effects of EVOO polyphenols, for therapeutic intervention aimed at increasing beneficial microbes. This will allow us to better understand how sex/gender affects health, leading to the development of tailored strategies to every single person.

## Figures and Tables

**Table 1:** Effect of extra virgin olive oil polyphenols on female and male gut microbiota.

Reference	Experimental Model	Healthy-Related Outcomes	Taxa Modifications in 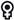 GM	Taxa Modifications in 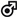 GM
Olalla et al., 2019 [168]	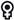 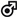 32 HIV patients; 50 g/day EVOO for 12 weeks	EVOO consumption was associated with a decrease in total cholesterol and an increase in the alpha-diversity of the GM in males	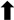 **Actinobacteria**, *Eggerthella lenta*; **Verrucomicrobia**, *Akkermansia muciniphila*; **Firmicutes**, *Clostridia*, *Ruminococcuss*, *Ruminococcus gnavus, Lachnospiraceae*; **Bacteroidetes**, *Parabacteroides* diastonis	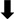 **Bacteroidetes**, *Prevotella copri, Prevotella* *stercorea*; **Actinobacteria**, *Bifidobacterium, Bifidobacterium bifidum*; **Firmicutes**, *Erysipelotrichaceae, Eubacterium*
Conterno et al., 2019 [163]	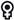 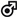 62 healthy hypercholesterolemic subjects; 90 g of olive pomace-enriched biscuits (containing 7.1 mg/100 g HT) for 8 weeks	Olive pomace-enriched biscuits reduced oxidized LDL cholesterol	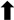 **Bacteroidetes**, *Bacteroides*, Rikenellaceae, Barnesiellaceae; **Verrucomicrobia**, *Akkermansia*; **Actinobacteria**, *Bifidobacterium*; **Proteobacteria**, *Enterobacteriaceae*	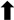 **Firmicutes**, *Eubacterium*; **Bacteroidetes**, *Prevotella*
Prieto et al., 2018 [160]	26 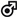 Swiss Webster ICR (CD-1) mice; HFD enriched with butter or with EVOO (527 mg/kg polyphenols) for 12 weeks	EVOO decreased plasmatic insulin level, blood pressure, and body weight		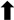 **Proteobacteria**, Sutterellaceae, *Marispillum* **Bacteroidetes**, *Mucilagini bacter dageonensis*
				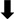 **Proteobacteria**, *Desulfovibrio*
Martinez et al., 2019 [159]	35 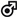 Swiss Webster ICR (CD-1) mice; HFD enriched with butter or OO or EVOO (527 mg/kg polyphenols) for 12 weeks	EVOO decreased plasmatic levels of insulin, glucose, and triglycerides		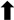 **Proteobacteria**, Sutterellaceae **Firmicutes**, *Erysipelotrichaceae*
				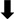 **Proteobacteria**, *Desulfovibrionaceae*, *Desulfovibrio, Helicobacteraceae*; *Tenericutes, Spiroplasmataceae*
Vezza et al., 2019 [169]	36 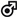 C57BL/6J mice; HFD diet or HFD with 1, 10 or 25 mg/kg/day OLE for 5 weeks	OLE reduced basal glycaemia, inflammatory status, and insulin resistance, and improved plasma lipid profile		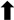 **Actinobacteria**, *Actinobacteria; Bacteroidetes, Bacteroidia*, *Cytophaga*; **Verrumicrobia**, *Akkermansia*
Wang et al., 2021 [170]	24 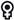 ICR mice; 100 mg/kg b/w Chinese olive fruit extracts (containing 10 wt.% HT) for 4 weeks	Olive fruit extract treatment improved the antioxidant capacity in mice, and reduced proinflammatory cytokine level	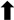 **Firmicutes**, *Colidextribacter;***Firmicutes/Bacteroidetes** ratio	
			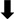 **Bacteroidetes**, *Alloprevotella*	
Liu et al., 2019 [171]	28 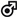 C57BL/6J mice; HFD diet or HFD with 50 mg/kg/day HT for 8 weeks	HT improves obesity and insulin resistance, reducing chronic inflammation		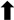 **Firmicutes**, *Lactobacillus johnsonii*
				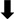 **Firmicutes**, Ruminococcaceae, Christensenellaceae, *Ruminiclostridium*; **Proteobacteria,***Desulfovibrio*; **Deferribacteres**; **Bacteroidetes**, *Rikenella*
Wang et al., 2019 [172]	15 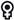 C57BL/6J mice; PM_2.5_-exposed or PM_2.5_-exposed treated with 50 mg/kg/day HT for 4 weeks	HT prevented visceral adipogenesis, oxidative stress, hepatic inflammation, and insulin resistance	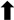 Verrucomicrobia, Akkermansia; Bacteroidetes, Porphyromonadaceae, Parabacteroides, Prevotellaceae UCG-001	
			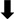 Actinobacteria, Bifidobacterium; Firmicutes, Ruminococcaceae, Mycoplasmataceae	
Li et al., 2022 [173]	30 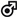 C57BL/6J mice; HFD or HFD with 0.2% (*w*/*w*) Tyr for 16 weeks	Tyr decreased plasma triacylglycerol, total cholesterol, and fasting glucose, promoting adipose thermogenesis		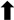 **Verrucomicrobia**; **Firmicutes**, Lachnospiraceae *Bacterium 28_4, Colidextrybacter*, *Clostridia, Oscillibacter*
				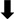 Firmicutes, Lactobacillus, Lachnospiraceae, Bacterium DW59
Rodriguez-Garcia et al., [174]	24 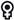 CD1 mice; coconut-HFD diet, sunflower HFD diet, or EVOO-HFD (333.4 g/kg) for 16 weeks	EVOO diet produced a GM anti-inflammatory environment, associated with protection against CRC development	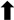 **Firmicutes***Lactococcus*; **Verrucomicrobia***Akkermansia*; **Firmicutes/Bacteroidetes** ratio	
			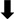 **Actinobacteria**, *Bifidobacterium*; **Proteobacteria***Neisseria*, *Pseudomonas* spp.; **Bacteroidetes Prevotella**; **Firmicutes** *Staphylococcus* spp., *Enterococcus gallinarum*	
Millman et al., 2020 [175]	20 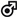 C57BL/6J mice; HFD, HFD-EVOO, or HFD-flaxseed oil for 10 weeks	EVOO enhanced gut immunity, and improved metabolic health in mice		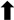 **Firmicutes**, Lachnospiraceae, *Allobaculum;* **Deferribacteres**, *Mucispirillum*, Coriobacteriaceae
				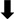 **Firmicutes**, *Clostridiales* spp.; **Bacteroidetes**, *S24-7* spp.
Zheng et al., 2021 [176]	15 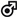 diabetic db/db mice; treatment with OLE (200 mg/kg) for 15 weeks	OLE ameliorated the advanced stage of T2D, decreasing fasting glucose, and improving glucose tolerance		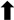 **Verrucomicrobia**, *Akkermansia*; **Deferribacteres**
				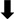 **Bacteroidetes**, *Prevotella*, *Odoribacter*, Parabacteroides; **Firmicutes**, *Ruminococcus*

Abbreviations: 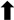, increase; 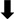, decrease; Bold, microbiota phylum; GM, gut microbiota; EVOO, extra virgin olive oil; OO, olive oil; Tyr, tyrosol; HT, hydroxytyrosol; OLE, oleuropein; HFD, high-fat diet; PM_2.5_, particular matter ≤2.5 μm; T2D, type 2 diabetes.
